# The Self-Administered Patient Satisfaction Scale for Primary Hip and Knee Arthroplasty

**DOI:** 10.1155/2011/591253

**Published:** 2011-01-10

**Authors:** N. Mahomed, Rajiv Gandhi, Lawrence Daltroy, J. N. Katz

**Affiliations:** ^1^Department of Surgery, Toronto Western Hospital, Toronto, ON, Canada; ^2^Harvard Medical School, Boston, MA, USA; ^3^Department of Epidemiology, Harvard School of Public Health, Boston, MA, USA

## Abstract

*Introduction*. The objective of this study was to develop a short self-report questionnaire for evaluating patient satisfaction with the outcome of hip and knee replacement surgery. *Methods*. This scale consists of four items focusing on satisfaction with the extent of pain relief, improvement in ability to perform home or yard work, ability to perform recreational activities, and overall satisfaction with joint replacement. This instrument does not measure satisfaction with process of care. The responses are scored on a Likert scale, with the total score ranging from 25 to 100 per question. The instrument was tested on 1700 patients undergoing primary total hip and total knee replacement surgery, evaluated preoperatively, at 12 weeks, and one year postoperatively. Psychometric testing included internal consistency, measured with Cronbach's alpha, and convergent validity, measured by correlation with changes in measures of health status between the preoperative, 12-week, and one-year evaluations. *Results*. The internal consistency (reliability) of the scale, measured by the Cronbach's alpha, ranged from 0.86 to 0.92. The scale demonstrated substantial ceiling effects at 1 year. The scale scores correlated modestly with the absolute SF-36 PCS and WOMAC scores (*ρ* = 0.56–0.63 and also with the WOMAC change scores (*ρ* = 0.38–0.46) at both 12-week and 1-year followups. *Conclusions*. This instrument is valid and reliable for measuring patient satisfaction following primary hip and knee arthroplasty and could be further evaluated for use with other musculoskeletal interventions.

## 1. Introduction

Total knee (TKA) and total hip arthroplasty (THA) are effective procedures for relieving pain and restoring function. Increasingly, patient satisfaction is being recognized as an important measure of health care quality [1]. 

Several studies have shown a discrepancy between surgeon and patient assessment of medical and surgical outcomes, particularly in assessing pain and function [2–5]. Surgeons generally focus on range of motion, alignment, and stability—objective measures—while patients are more concerned with overall functionality of the joint—subjective measures [6, 7]. Moreover, the outcome of satisfaction likely stretches beyond improved mobility and pain relief, but rather encompasses other factors such as fulfillment of preoperative expectations [8, 9]. A complete evaluation of total joint replacement (TJR) outcomes should therefore include clinical measures, generic and disease-specific functional health outcomes scales, and patient satisfaction. 

There are a number of published scales on patient satisfaction, but these focus on issues of process of care, such as the office environment, the patient-physician interaction, or general satisfaction with care [10–12]. Although many authors have reported on patient satisfaction after TJR, we are unaware of any validated self-report scale that evaluates patient satisfaction with the results of musculoskeletal treatment outcomes. Knowledge of modifiable factors predicting patient dissatisfaction following surgery, such as expectations or comorbidity, would be valuable for physicians and patients in order to improve satisfaction with surgical outcomes. 

The goal of this study was to develop and perform psychometric testing (internal consistency and convergent validity) for a short self-report questionnaire evaluating patient satisfaction with the outcome of hip and knee arthroplasty. The Self-Administered Patient Satisfaction Scale (SAPS) was designed to be used in conjunction with other clinical measures and functional health status instruments to evaluate the results of hip and knee arthroplasty. We hypothesized that individuals who experienced less pain and better functioning and greater improvements in these areas would likewise show greater satisfaction with their surgery [8, 13].

## 2. Methods

### 2.1. Scale Description

Four items, as determined by literature review and an expert consensus panel—rheumatologists, an orthopaedic surgeon, and a behavioral scientist—were chosen reflecting various facets of patient functioning most affected by TJR. The items include patients' overall satisfaction with surgery, the extent of pain relief, the ability to perform home or yard work, and the ability to perform recreational activities. Items are scored on a 4-point Likert scale with response categories consisting of very satisfied (100 points), somewhat satisfied (75 points), somewhat dissatisfied (50 points), and very dissatisfied (25 points). The scale score is the unweighted mean of the scores from the individual items, ranging from 25 to 100 per item (with 100 being most satisfied). 

*Face validity* was assessed by having the scale reviewed by a panel of independent experts in the field of rheumatology and orthopedics.

### 2.2. Instrument Testing

As part of our registry, patients are recruited from a single Canadian academic institution, the Toronto Western Hospital, prior to undergoing primary TKA and THA. For this study, we included patients aged 18 years or above with a diagnosis of primary or secondary osteoarthritis (OA), or inflammatory arthritis. All patients gave informed consent to participate in this study. All data were collected by an independent assessor not involved in the patient's medical care. The study protocol was approved by the local ethics committee.

### 2.3. Data Collection

Baseline demographic data of age, gender, body mass index (BMI), and ethnicity were collected by patient self-report at the time of surgery. Ethnicity was recorded under the categories of White/European, Black, Asian, or Aboriginal as defined by the United States Census [14]. Asian refers to individuals who classified themselves as South Asian (India, Pakistan, Bangladesh and Sri Lanka) or East Asian (China, Japan, Taiwan, Korea).

Functional status was assessed at baseline, 12-week and 1-year followup using the Western Ontario McMaster University Osteoarthritis Index (WOMAC) [15], and the Medical Outcomes Short Form 36 (SF 36) Physical Component Score (PCS) [16, 17]. For ease of presentation, the WOMAC scores were rescaled from 0–100 with high scores representing better function and pain relief. The SF 36 is the most common generic health scale used in a joint arthroplasty population [18]. The WOMAC index has become the standard scale adopted by the American Academy of Orthopaedic Surgeons and the Council Of Musculoskeletal Societies for the assessment of joint (hip and knee) functional outcomes. Patient satisfaction was assessed at the same followup points using the SAPS.

#### 2.3.1. Scale Characteristics

**Reliability**

The distributions of scale scores were examined with tests of normality including skewness and kurtosis and graphically with histograms. We tested for floor and ceiling effects by calculating the percentage of respondents scoring at the lowest and the highest scale levels, respectively. The internal consistency (reliability) of the scale was evaluated using the Cronbach's alpha coefficient. The Cronbach's alpha calculates how well a set of variables correlate with one another and with the aggregate scale score [19]. It is used to assess the capacity of the scale to measure a unidimensional concept. Values of 0.7 or greater are considered acceptable, values greater than 0.8 are considered good, and greater than 0.9 are considered excellent [19].

**Convergent Validity**

We compared the results of the satisfaction scale against the absolute total WOMAC score and SF 36 PCS as well as against the change in total WOMAC score at both 12-week and 1-year followup. The change score represents the improvement in pain and function from surgery each reported patient calculated as the difference between the followup score and the baseline score. The WOMAC change score has been shown by others to be related to patient-reported satisfaction with TJR [13]. Spearman's rank correlation coefficient was used as not all data was normally distributed.

Separate statistical analyses were conducted for hip and knee patients. All analyses were completed with SPSS version 13.0 (SPSS, Chicago, IL, USA). All reported p values are 2-tailed with an alpha of 0.05.

## 3. Results

The validity and internal consistency (reliability) of the scale were assessed on complete data from 843 hip arthroplasty patients and 857 knee arthroplasty patients. This represents a response rate of 1700/2000 (85%). The demographic data, functional outcomes, and satisfaction scores of these groups are presented in Table 1. The mean age of the group was 65.2 years (range 19–88, SD 11.3) while 44.4% were males. The knee arthroplasty patients, on average, were a few years older than the hip arthroplasty patients with a greater percentage of females. The demographic data of responders and nonresponders was not clinically or statistically different (data not shown).

### 3.1. Response Patterns

The majority of responses were in the very satisfied or somewhat satisfied categories. More patients were satisfied with pain relief compared to improvement in their ability to do work or recreational activities [18, 20]. All scale scores demonstrated a negative skew for both hip and knee patient populations, indicating that respondents' scores were clustered toward the positive end of the health spectrum.

Table 2 presents the distribution of responses for each item in the scale. Substantial ceiling effects (62% and 52%) were found for the 1-year hip and knee scale scores, respectively. Modest ceiling effects (39% and 30%) were found for the 12 week hip and knee scale scores, respectively. There was minimal evidence of floor effects for both hip and knee patients as less than 3% scored at the lowest end of the scale.

### 3.2. Scale Characteristics: Reliability

The 12 week and 1-year cronbach's alpha (reliability) coefficients for satisfaction scores are given in Table 3.

### 3.3. Scale Characteristics: Convergent Validity

#### 3.3.1. Hips

The 12 week Spearman's correlation coefficient for total WOMAC scores and satisfaction scores was 0.57, *P* < .001. At 1-year followup, the correlation coefficient was 0.60, *P* < .0001. For the outcome of the total WOMAC change score, the Spearman's correlation coefficients at 12-week and 1-year followup were 0.43, *P* < .001 and 0.44, *P* < .001, respectively.

The 12 week Spearman's correlation coefficient between the SF36 PCS and satisfaction scores was 0.55, *P* < .001. At 1-year followup, the correlation coefficient was 0.55, *P* < .0001. For the outcome of the PCS change score, the Spearman's correlation coefficients at 12-week and 1-year followup were 0.46, *P* < .001 and 0.46, *P* < .001, respectively.

#### 3.3.2. Knees

The 12 week Spearman's correlation coefficient for total WOMAC scores and satisfaction scores was 0.61, *P* < .001. At 1-year followup, the correlation coefficient was 0.64, *P* < .0001. For the outcome of the total WOMAC change score, the Spearman's correlation coefficients at 12-week and 1-year followup were 0.41, *P* < .001 and 0.38, *P* < .001, respectively.

The 12 week Spearman's correlation coefficient between the SF36 PCS scores and satisfaction scores was 0.53, *P* < .001. At 1-year followup, the correlation coefficient was 0.57, *P* < .0001. For the outcome of the PCS change score, the Spearman's correlation coefficients at 12-week and 1-year followup were 0.38, *P* < .001 and 0.41, *P* < .001, respectively.

**Figure 1:**
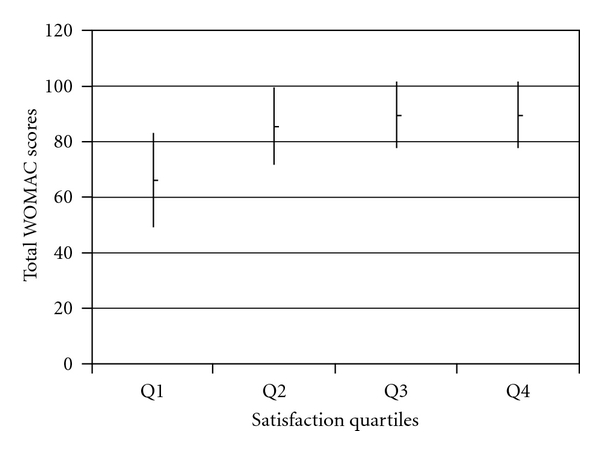
Mean 1-year total WOMAC scores (with SD) compared across quartiles of 1-year satisfaction scores for hip patients.

**Figure 2:**
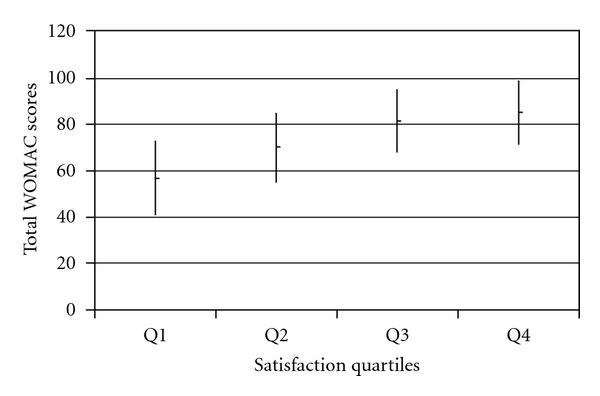
Mean 1-year total WOMAC scores (with SD) compared across quartiles of 1-year satisfaction scores for knee patients.

Figures 1 and 2 demonstrate the relationship between the quartiles of satisfaction scores and mean total WOMAC scores for hip and knee patients at 1-year, respectively. For the hip patients, the mean WOMAC scores for quartiles 2 to 4 are similar due to the ceiling effect as satisfaction scores were high across those quartiles. The knee patients demonstrated more of a graded response between quartiles of satisfaction and mean WOMAC scores indicating less of a ceiling effect.

## 4. Discussion

The assessment of TJR outcomes has evolved from focusing primarily on clinical outcomes, to patient report measures and patient satisfaction. It has been estimated that between 9 and 30% of patients may be dissatisfied following surgery and thus a greater understanding of the determinants of patient satisfaction may help to improve subjective outcomes [8, 21–23]. This short four-item satisfaction scale is a reliable (high internal consistency) and valid instrument for measuring satisfaction with the outcome of TJR. The distribution of responses for each item revealed that patients were more satisfied with improvement in pain than function. This correlates with clinical experience and the published literature, where pain relief is more reliably achieved than improvement in function with TJR [18]. One group showed that 73% of patients were very satisfied with pain relief, but only 50% were very satisfied with their ability to perform leisure activities following TKA [20]. Other potential reasons for patient dissatisfaction with surgery may be poor mental health, unfulfilled expectations, the patient-surgeon relationship, or length of the incision [8, 9, 22, 24–27].

Further, we found that the total satisfaction scores were greater at 12-week and 1-year following THA as compared to TKA, which is consistent with the literature that shows greater satisfaction and health improvement following hip arthroplasty [28, 29]. Hip arthroplasty consistently demonstrates superior functional outcomes as compared to the knee, potentially because the ball and socket design of a hip joint is easier to replicate with metallic implants as compared to a more complicated hinge joint such as the knee.

The scale has a broad distribution of scores with substantial ceiling effects at 1-year. Similar floor/ceiling effects are seen in widely used scale such as the SF 36 [30–32] and in other satisfaction scales [33]. The instrument should take approximately 2 minutes to complete. It could therefore be added to other outcome instruments to more completely evaluate the results of TJR without significantly increasing respondent burden. This scale differs from others in the literature in that it only focuses on satisfaction with the outcome of an intervention rather than the process of care [10–12].

We found a slightly better correlation for the absolute scores of the WOMAC index as compared to the WOMAC change scores at both 12-week and 1-year followup. This suggests that satisfaction following TJR may be more predicted by the final status reached rather than the relative benefit gained from the surgery. Moreover, it suggests that although patient satisfaction is related to improvement in pain and function, these domains are not directly correlated. Thus the satisfaction scale measures a different but interrelated domain. 

Limitations of this study include the use of the Likert response scale that yields ordinal rather than interval data. Caution with parametric analysis is thus required. However, the Cronbach's alpha coefficient indicated good reliability for summing the individual items into a summary score. Second, we did not assess test-retest reliability due to the nature of the existing study designs of the two cohorts used. Third, the scale was not validated on a cohort of revision arthroplasty patients, and this would be required prior to its use in this population. Fourth, although the scale asks about general satisfaction “with results of surgery”, it would require validation on other musculoskeletal surgical interventions before its use could be generalized. 

In conclusion, this satisfaction scale may be used in conjunction with other outcome instruments to more comprehensively evaluate the results of primary hip and knee replacement surgery. It has been shown that patient satisfaction following surgery does not always correlate with surgeon assessments [3, 6] and this scale provides a simple instrument to explore the complex relationships between patient baseline pain, functioning, expectations of surgery, and satisfaction with outcome. Identification of any modifiable risk factor for patient dissatisfaction with surgery presents an opportunity of improving patients perceived outcomes.

## Figures and Tables

**Table 1:** Demographic data, satisfaction scores, and total WOMAC scores for hip and knee replacement patients used in scale assessment.

	Hips	Knees
	*n* = 843	*n* = 857
Mean age (SD)	64.8 (12.6)	67.3 (9.9)
% Males	46%	36.8%
Mean BMI kg/m^2^ (SD)	27.4 (5.5)	30.4 (6.5)
% White	76%	71%

Mean/median 12 week		
Satisfaction	90.6/100	83.4/87.5
score (range,SD)	(25–100,13.8)	(25–100,17.2)

Mean/median 12 week		
total WOMAC	76.8/81	68.9/70
Score (range,SD)	(20–100,17.2)	(4–100,18.2)

Mean/median 12 week		
SF36 PCS	40.1/39.8	36.8/35.8
scores (range,SD)	(11.5–65.1,10.1)	(13.6–61.6,9.2)

Mean/median 1 year		
Satisfaction	92.4/87.5	84.2/100
score (range,SD)	(25–100,13.5)	(25–100,19.0)

Mean/median 1-year total		
WOMAC	82.4/88	72.7/76
scores (range,SD)	(15–100,16.9)	(8–100,18.9)

Mean/median 1-year SF36		
PCS scores (range,SD)	44.2/44.6 (16.6–67.6,11.0)	34.4/38.7 (14.7,68.5,10.5)

**Table 2:** Percentage distribution for responses for each item.

	Hips		Knees	
	12 wks (%)	1 year (%)	12 wks (%)	1 year (%)
How satisfied are you with the results of your surgery ?				
Very satisfied	83.4	83.0	64.1	65.8
Somewhat satisfied	13.4	13.6	28.7	22.2
Somewhat dissatisfied	1.8	2.1	5.1	8.1
Very dissatisfied	1.4	1.3	2.1	3.9

How satisfied are you with the results of your surgery for improving your pain?				
Very satisfied	84.4	86.9	63.4	67.6
Somewhat satisfied	12.3	10.7	27.7	21.9
Somewhat dissatisfied	2.3	1.6	5.8	7.3
Very dissatisfied	1.0	0.8	3.2	3.2

How satisfied are you with the results of surgery for improving your ability to do home or yard work?				
Very satisfied	61.0	71.7	40.0	50.2
Somewhat satisfied	30.3	20.1	45.7	33.9
Somewhat dissatisfied	5.0	5.9	9.8	10.7
Very dissatisfied	3.7	2.3	4.4	5.2

How satisfied are you with the results of surgery for improving your ability to do recreational activities?				
Very satisfied	53.6	65.8	34.0	42.8
Somewhat satisfied	34.7	24.7	46.9	36.2
Somewhat dissatisfied	7.1	6.9	12.8	13.1
Very dissatisfied	4.6	2.6	6.2	7.9

**Table 3:** Cronbach's alpha coefficients for internal consistency (reliability) of the satisfaction scale.

	Hips	Knees
12-week satisfaction	0.86	0.91
1-year Satisfaction	0.91	0.92
